# Supplementary material for: Distinct pulmonary and systemic effects of dexamethasone in severe COVID-19
Source: Nat Commun. 2024 Jun 28;15:5483. doi: 10.1038/s41467-024-49756-2 (PMC11213873; doi:10.1038/s41467-024-49756-2)
Supplement: Supplementary file 1 — Supplementary Information [file 41467_2024_49756_MOESM1_ESM.pdf]

Distinct pulmonary and systemic effects of dexamethasone in severe COVID-19

Supplementary Information

Table S1 | Demographics table at admission (unless specified otherwise).

Variable	Category	All (N=43)	No Dexamethasone (N=16)	Dexamethasone (N=27)	No Dex vs. Dex
Age	(years)	58.0 (45.5-68.5)	50.5 (40.5-64.2)	62.0 (53.0-70.5)	*P*=.05
Sex at birth	Male	30 (69.8%)	11 (68.8%)	19 (70.4%)	*P=1*
BMI		33 (30.3-37.5)	32.7 (28.7-37)	33.5 (30.3-38.2)	*P=.76*
Race	Asian	2 (4.7%)	2 (12.5)	0 (0.0)	*P=.17*
	Black / African American	2 (4.7%)	0 (0.0%)	2 (7.4%)	
	Native Hawaiian / Other Pacific Islander	0 (0.0%)	0 (0.0%)	0 (0.0%)	
	Other / Multiple Races	31 (72.1%)	12 (75.0%)	19 (70.4%)	
	White	8 (18.6%)	2 (12.5%)	6 (22.2%)	
Ethnicity	Hispanic / Latino	27 (62.8%)	10 (62.5%)	17 (63.0%)	*P=1*
Mean arterial pressure	(mmHg)	97.3 (84.7-105.5)	95.0 (81.8-100.7)	98.0 (85.7-106.2)	*P=.39*
Diastolic blood pressure	(mmHg)	77.0 (69.0-84.0)	79.0 (65.2-86.8)	76.0 (70.5-82.0)	*P=.66*
Systolic blood pressure	(mmHg)	134.0 (116.0-145.5)	119.0 (106.0-136.5)	140.0 (122.5-150.0)	*P=.03*
FiO2		0.2 (0.2-0.5)	0.2 (0.2-0.4)	0.2 (0.2-0.6)	*P=.59*
P/F ratio at Day 0		97 (68-150)	97 (71-155)	95 (68-147)	*P=.72*
Oxygen saturation	(%)	90 (85-96)	93 (88-96)	90 (84-95)	*P=.39*
Heart rate	(beats per minute)	108 (87-123)	112 (85-125)	105 (91-121)	*P=.35*
Respiratory rate	(breaths per minute)	25 (22-30)	25 (24-32)	26 (21-30)	*P=.76*
Temperature	(Celsius)	36.8 (36.7-37.5)	36.8 (36.7-37.2)	36.8 (36.7-37.6)	*P=.67*
Neutrophil/WBC		0.85 (0.79-0.90)	0.85 (0.83-0.90)	0.85 (0.77-0.91)	*P=.78*
N antigen	(pg/mL)	481.2 (5.3-4510.8)	24.5 (2.1-2162.5)	958.7 (335.5-4887.5)	*P=.04*
Time between first Dex and D0 sample	(days)	/	/	2 (1-2.5)	/
Remdesivir	Yes	32 (74%)	5 (31%)	27 (100%)	*P<.001*
Discharge status	In-hospital death	10 (23.3%)	2 (12.5%)	8 (29.6%)	*P=.43*
	Activity limitations and/or O2 requirements	23 (53.5%)	10 (62.5%)	13 (48.1%)	
	No limitations and no O2 requirements	10 (23.3%)	4 (25.0%)	6 (22.2%)	
Ventilator-free days		7.0 (2.0-18.5)	12.0 (0.0-18.2)	5.0 (2.5-18.0)	*P=.91*
Alive	Yes	32 (74.4%)	14 (87.5%)	18 (66.7%)	*P=.25*

No Dex. vs. Dex differences tested using two-sided Wilcoxon rank-sum tests for continuous variables and Chi-squared tests for categorical variables. No adjustment of p-values was performed here.

Table S2 | Measured cytokine biomarkers.

Cytokine	Cytokine full name
Ang-1	Angiopoietin 1
Ang-2	Angiopoietin 2
ICAM-1	Intercellular adhesion molecule 1
IFN-gamma	Interferon gamma
IL-10	Interleukin 10
IL-18	Interleukin 18
IL-6	Interleukin 6
IL-8	Interleukin 8
IP-10	Interferon gamma-induce protein 10
MMP-8	Matrix metalloproteinase 8
PAI-1	Plasminogen activator inhibitor 1
Protein C	/
RAGE	Receptor for advanced glycation end-products
SP-D	Surfactant protein D
Thrombomodulin	/
TNR R1	Tumor necrosis factor receptor 1
TREM-1	Triggering receptor expressed on myeloid cells 1
VEGF	Vascular endothelial growth factor

Table S3 | Significant gene expression counts per cell type across compartments.

Cell type	Both compartments	TA only	WB only
CD4 T	1	0	177
CD8 T	1	4	38
Monocytes	8	13	176
Neutrophils	14	9	92
NK	0	11	19
other	0	0	37
Tregs	3	84	50

Number of significantly different genes per cell type using MAST (two-sided; BH-adj. p-value < 0.1 & $|log_2FC| > 0.25$). N = 7 Dex, 3 NoDex for WB, and N = 10 Dex, 7 NoDex or TA.
TA = tracheal aspirate. WB = whole blood. Both compartments = both WB and ETA samples.

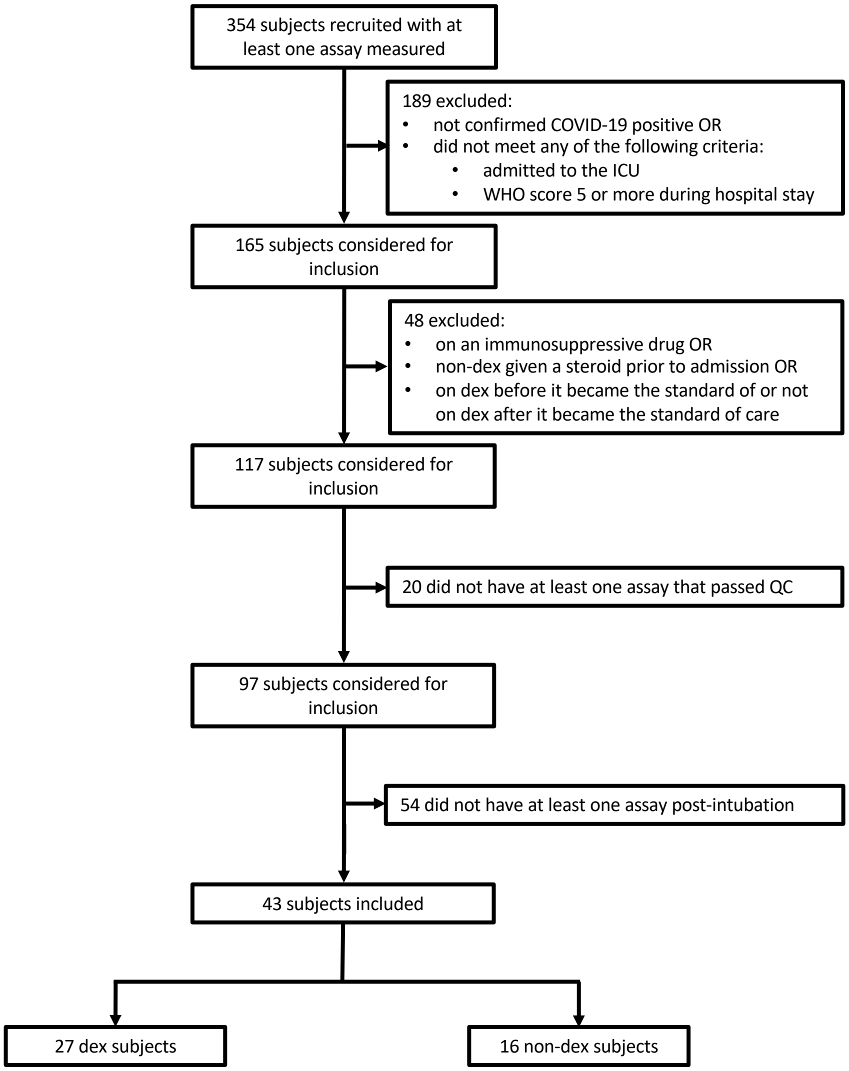

Figure S1 | Consort chart.

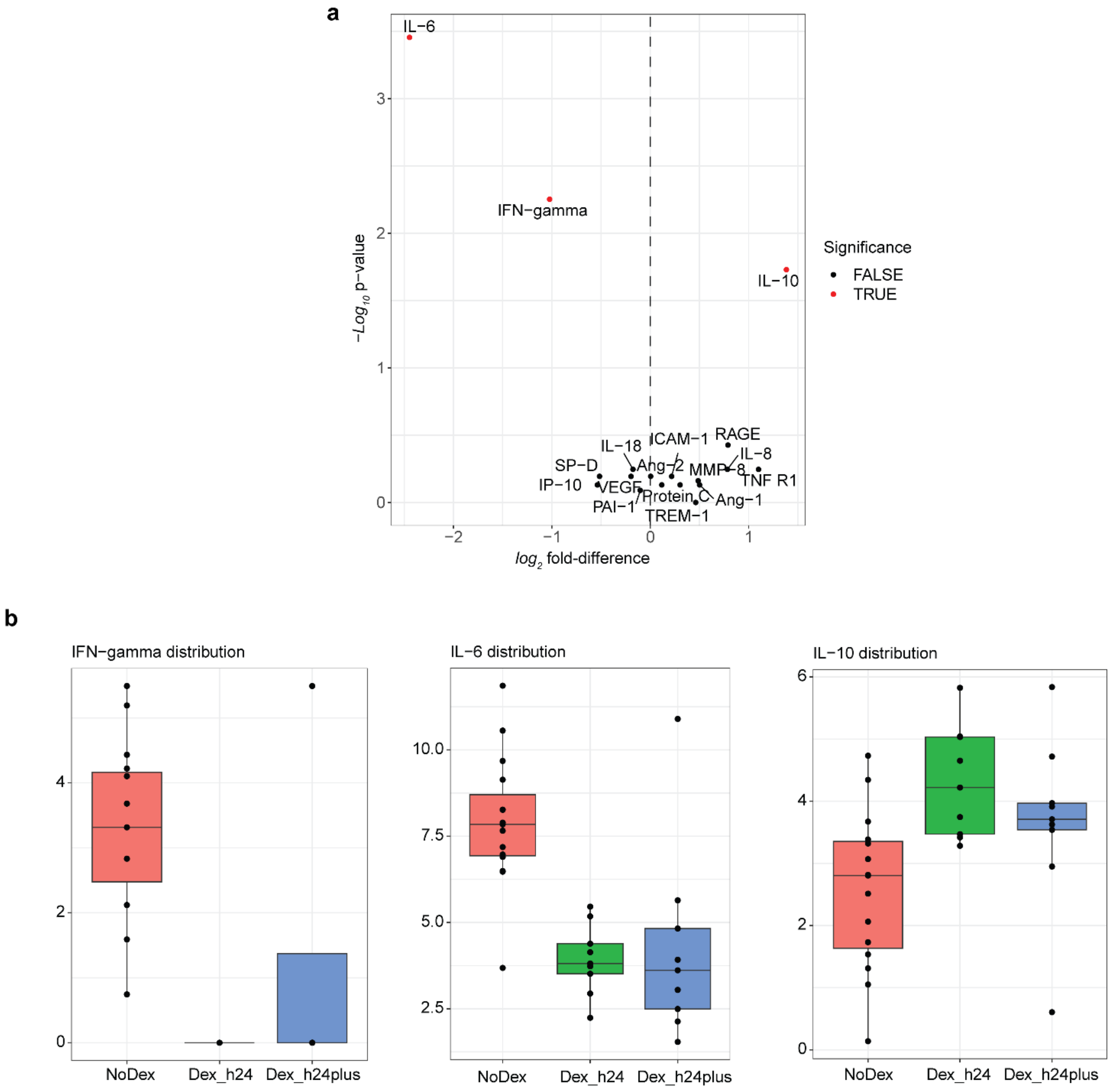

Figure S2 | Differences in cytokine expression. a, Volcano plot of cytokines comparing Dex (right > 0) and NoDex (left < 0), colored by significance (red; two-sided Wilcoxon rank-sum test, BH-adjusted p-value < 0.1). N = 23 Dex, N = 15 NoDex, for 18 cytokines; day 0 of hospitalization. **b,** Cytokine differences, stratified by time between first dexamethasone dose and sample collection. The median, first and third quartiles, and 1.5*interquartile range distance from the quartiles are represented using the center mark, hinges, and whiskers, respectively. N = 23 Dex, N = 15 NoDex.

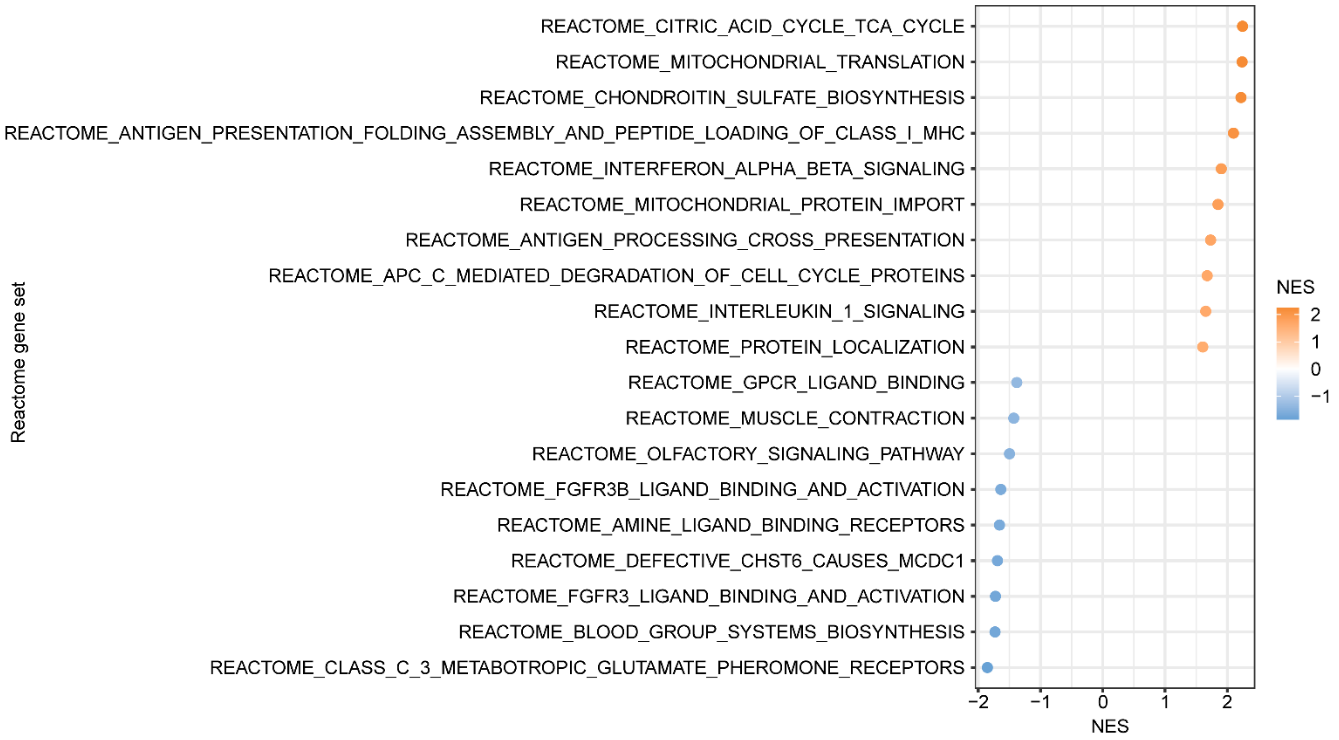

Figure S3 | Gene set enrichment of bulk RNA-seq from PBMC. Gene set enrichment plot of 19 most significant – top 10 for Dex (orange) and top 9 for NoDex (blue) – Reactome terms, based on differential gene expression results. Only significant terms with a BH-adjusted p-value <.1 were selected (one-sided test based on a modified Kolmogorov-Smirnov statistic). N = 10 Dex, N = 11 NoDex.

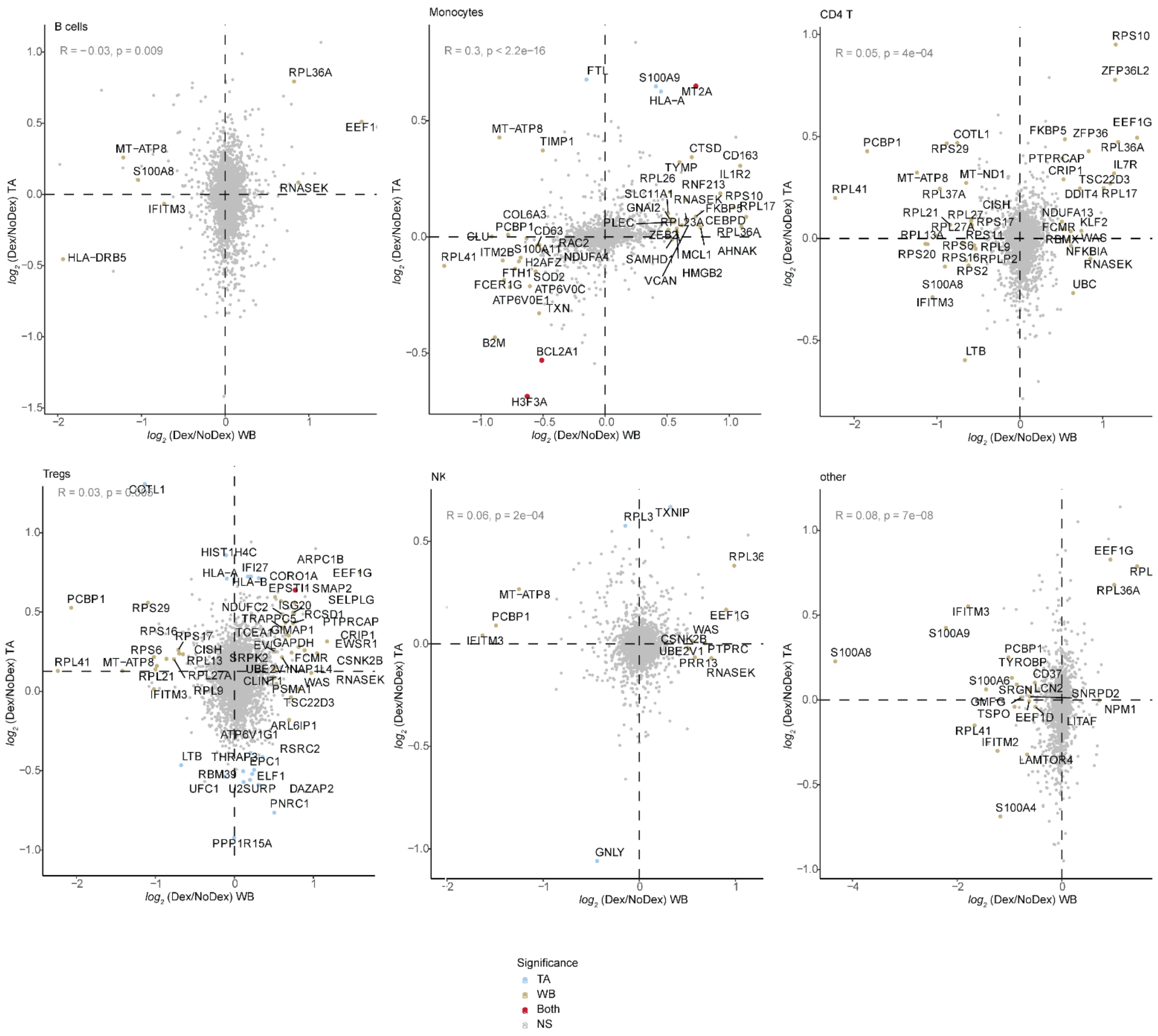

Figure S4 | Cross tissue differential gene expression. *log_2* fold-difference in gene expression of Dex and NoDex in TA (y-axis; N = 10 Dex, 7 NoDex) v. blood (x-axis; N = 7 Dex, 3 NoDex) plotted for additional cell types not shown in Figure 3. Significant genes in TA only (blue), blood only (brown), both compartments (red) are shown (BH-adj. p-value < 0.1 & | *log_2* fold-difference| > 0.5). Spearman's correlation R value shown between the two compartments.

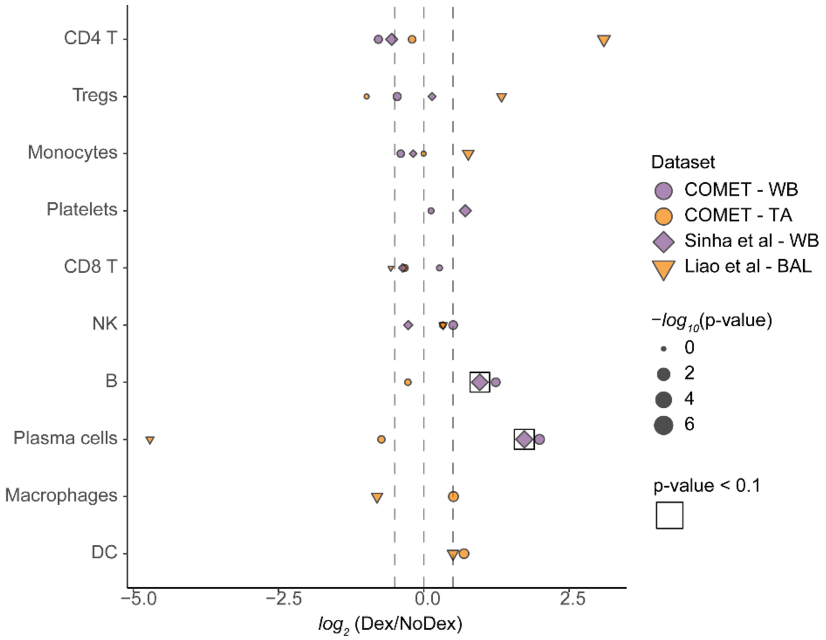

Figure S5 | Immune cell frequencies quantified and compared between Dex and NoDex samples. X-axis shows *log_2* fold-difference of Dex compared to NoDex in whole blood (purple circle); TA (orange circle); a blood validation set (Sinha et al, purple diamond); a lung validation set (bronchial alveolar lavage; Liao et al, orange triangle). Significance shown by boxes. The size of each shape corresponds to *-log_{10}* p-value calculated using the Wilcoxon rank-sum test. Ns reported in Figure S8.

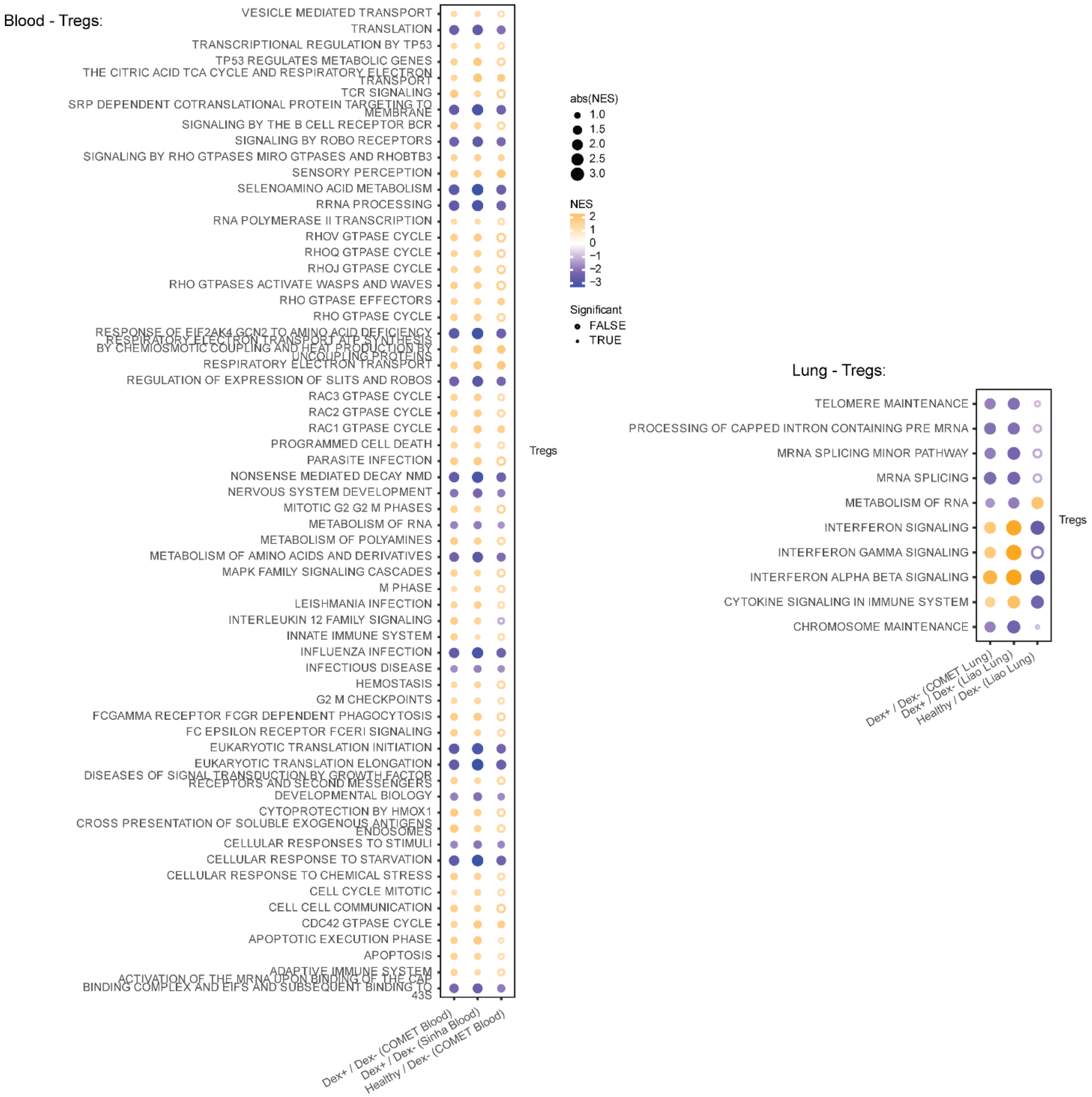

Figure S6 | Gene set enrichment of Tregs in blood and lung. Net enrichment scores from gene set enrichment analysis in blood and lung shown for Tregs (remaining cell types shown in Figure 4). Fold differences are shown for dexamethasone-treated samples (Dex), or healthy control samples, all relative to the NoDex samples within that dataset. Orange shows up in Dex or healthy relative to NoDex COVID-19 samples, bule shows down in Dex or healthy. Datasets represented are from COMET (whole blood, TA), Sinha et al (blood) and Liao et al (BAL). Significance was determined using a BH-adjusted p-value threhsold of 0.1 with a one-sided test based on a modified Kolmogorov-Smirnov statistic. Ns reported in Figure S8.

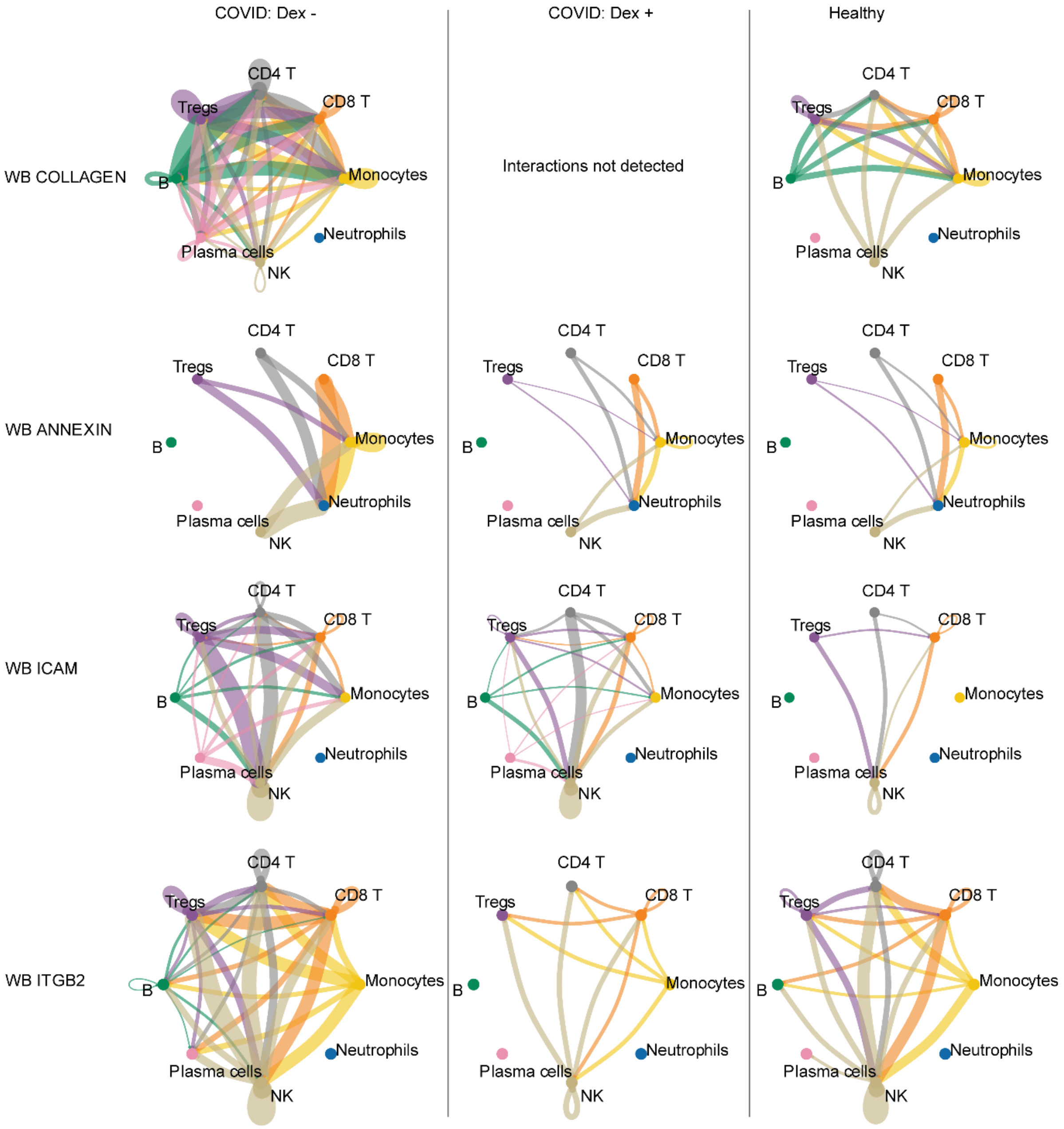

Figure S7 | Whole blood cell interactions using CellChat. CellChat interaction networks for COLLAGEN, ANNEXIN, ICAM and ITGB2 interactions shown comparing NoDex (left), and Dex (middle) patients, and healthy controls (right) for COMET whole blood dataset. Line thickness represents predicted strength of the interaction. Ns reported in Figure S8.

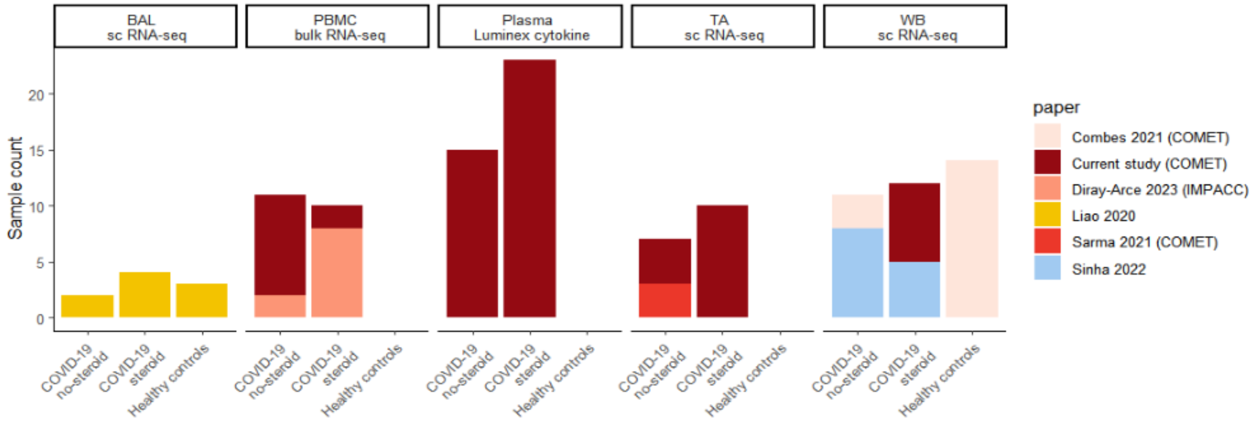

Figure S8 | Data sources for each analysis. The current study uses data from prior publications as well as presents new data that has not been made available prior to this work. Data sources are summarized by modality. For a sample-level report as well as associated accession numbers see Supplementary Data File 2.
