# Supplementary material for: Distinct pulmonary and systemic effects of dexamethasone in severe COVID-19
Source: Nat Commun. 2024 Jun 28;15:5483. doi: 10.1038/s41467-024-49756-2 (PMC11213873; doi:10.1038/s41467-024-49756-2)
Supplement: Supplementary file 3 — Description of Additional Supplementary Files [file 41467_2024_49756_MOESM3_ESM.pdf]

Description of Additional Supplementary Files

Supplementary Data 1: Full differential gene expression results (MAST) for COMET, Sinha et al, and Liao et al comparisons.

Supplementary Data 2: List of samples used in the analyses presented here along with their metadata and accession numbers.
