# Supplementary material for: Distinct pulmonary and systemic effects of dexamethasone in severe COVID-19
Source: Nat Commun. 2024 Jun 28;15:5483. doi: 10.1038/s41467-024-49756-2 (PMC11213873; doi:10.1038/s41467-024-49756-2)
Supplement: Supplementary file 6 — Reporting Summary [file 41467_2024_49756_MOESM6_ESM.pdf]

nature portfolio

Corresponding author(s): Gabriela Fragiadakis
Last updated by author(s): Apr 29, 2024

Reporting Summary

Nature Portfolio wishes to improve the reproducibility of the work that we publish. This form provides structure for consistency and transparency in reporting. For further information on Nature Portfolio policies, see our Editorial Policies and the Editorial Policy Checklist.

Statistics

For all statistical analyses, confirm that the following items are present in the figure legend, table legend, main text, or Methods section.

n/a	Confirmed	
☐	☒	The exact sample size (*n*) for each experimental group/condition, given as a discrete number and unit of measurement
☐	☒	A statement on whether measurements were taken from distinct samples or whether the same sample was measured repeatedly
☐	☒	The statistical test(s) used AND whether they are one- or two-sided<br>*Only common tests should be described solely by name; describe more complex techniques in the Methods section.*
☐	☒	A description of all covariates tested
☐	☒	A description of any assumptions or corrections, such as tests of normality and adjustment for multiple comparisons
☒	☐	A full description of the statistical parameters including central tendency (e.g. means) or other basic estimates (e.g. regression coefficient) AND variation (e.g. standard deviation) or associated estimates of uncertainty (e.g. confidence intervals)
☐	☒	For null hypothesis testing, the test statistic (e.g. *F*, *t*, *r*) with confidence intervals, effect sizes, degrees of freedom and *P* value noted<br>*Give P values as exact values whenever suitable.*
☒	☐	For Bayesian analysis, information on the choice of priors and Markov chain Monte Carlo settings
☒	☐	For hierarchical and complex designs, identification of the appropriate level for tests and full reporting of outcomes
☒	☐	Estimates of effect sizes (e.g. Cohen's *d*, Pearson's *r*), indicating how they were calculated

Our web collection on statistics for biologists contains articles on many of the points above.

Software and code

Policy information about availability of computer code

Data collection	Clinical data was abstracted from the electronic medical records and stored in a Redcap database.
Data analysis	All statistical analysis was completed using R version 4.2.

For manuscripts utilizing custom algorithms or software that are central to the research but not yet described in published literature, software must be made available to editors and reviewers. We strongly encourage code deposition in a community repository (e.g. GitHub). See the Nature Portfolio guidelines for submitting code & software for further information.

Data

Policy information about availability of data

All manuscripts must include a data availability statement. This statement should provide the following information, where applicable:

- Accession codes, unique identifiers, or web links for publicly available datasets
- A description of any restrictions on data availability
- For clinical datasets or third party data, please ensure that the statement adheres to our policy

The data files used to produce the results reported in this article are available on Gene Expression Omnibus (GEO), dbGaP or Dryad. The computable matrix of the plasma cytokine data is deposited at Dryad [(https://doi.org/10.7272/Q6MS3R18]doi:10.7272/Q6MS3R18). The raw and processed sequencing data for COMET samples used here is available at GEO under GSE237180 [https://www.ncbi.nlm.nih.gov/geo/query/acc.cgi?acc=GSE237180] SuperSeries. The FASTQ files and processed data files for the bulk RNA-seq data are available at GEO (GSE237109), dbGaP (phs002686.v1.p1; [https://www.ncbi.nlm.nih.gov/projects/gap/cgi-bin/study.cgi?study_id=phs002686.v1.p1])) and at ImmPort (SDY1760; [https://www.immport.org/shared/study/SDY1760]). The cellranger-processed raw feature-

barcode matrices for tracheal aspirate and whole-blood are available at GEO (GSE236030), and the associated raw FASTQ files for 10X libraries have been deposited in the Sequence Read Archive (SRA). A subset of the whole-blood data published in our previous article10 was obtained from GSE163668 [https://www.ncbi.nlm.nih.gov/geo/query/acc.cgi?acc=GSE163668] (HS1 and HS2 from GSM4995425, HS50 from GSM4995430, and the healthy controls from GSM4995449- GSM4995462) The whole-blood data reported in Sinha et al was secured from GSE157789 [https://www.ncbi.nlm.nih.gov/geo/query/acc.cgi?acc=GSE157789] and the BAL data in Liao et al from GSE145926 [https://www.ncbi.nlm.nih.gov/geo/query/acc.cgi?acc=GSE145926]. The accession numbers and sample metadata are included in Supplementary File 2.
Source data are provided with this paper.

Research involving human participants, their data, or biological material

Policy information about studies with human participants or human data. See also policy information about sex, gender (identity/presentation), and sexual orientation and race, ethnicity and racism.

Reporting on sex and gender	Differential expression models were adjusted for sex assigned at birth. Both clinical sites separately collect data on gender identity, which was recorded separately in the research database. No sex-based analyses were performed as statistical power would have been insufficient.
Reporting on race, ethnicity, or other socially relevant groupings	The cohort studied in this paper is representative of patients admitted to the medical intensive care units in the San Francisco Bay Area during the COVID-19 pandemic. Data on self-reported race and ethnicity are included in Supplementary Table 1, but was not used to adjust statistical analyses.
Population characteristics	The patient population was 70% male with an average age of 58. 16 patients were enrolled before dexamethasone was the standard of care for severe COVID and 27 subjects were admitted after dexamethasone was the standard of care.
Recruitment	Patients admitted to UCSF Medical Center or San Francisco General Hospital who tested positive for COVID-19 after April 2020 were eligible for enrollment in the COMET cohort. This study focused on the subset of patients who were mechanically ventilated with acute respiratory distress syndrome due to COVID-19.
Ethics oversight	The COMET study was approved by the UCSF Institutional Review Board (IRB #: 20-30497).

Note that full information on the approval of the study protocol must also be provided in the manuscript.

Field-specific reporting

Please select the one below that is the best fit for your research. If you are not sure, read the appropriate sections before making your selection.

☒ Life sciences ☐ Behavioural & social sciences ☐ Ecological, evolutionary & environmental sciences

For a reference copy of the document with all sections, see nature.com/documents/nr-reporting-summary-flat.pdf

Life sciences study design

All studies must disclose on these points even when the disclosure is negative.

Sample size	Subjects were selected from an observational cohort of patients admitted to two academic centers. We included all eligible subjects with COVID-19 ARDS who either received no immunosuppression or received dexamethasone for COVID ARDS. 27 patients who received at least one dose of 6mg dexamethasone at the time of initial biosampling (Dex) and 16 patients who did not received dexamethasone (NoDex) prior to specimen collection. These sample sizes were adequate to detect moderate changes in gene expression in bulk RNA and scRNAseq analyses.
Data exclusions	We excluded patients who received standard steroids for an indication other than COVID-19 and those who received other immunosuppresive drugs (e.g. tocilizumab, baricitinib).
Replication	We repeated our single-cell analyses in independent analyses of publicly available data from two external cohorts. For the other data types, no datasets were available to perform replication analyses.
Randomization	This was an observational study of patients admitted to two academic medical centers. Treating physicians prescribed steroids as part of clinical care. At the study sites, steroids were not routinely prescribed for COVID prior to the publication of the RECOVERY trial in July 2020.
Blinding	Investigators were not blinded to study group during the final analyses. Dependent variables in this study (protein concentrations, sequencing) were measured using standardized methods.

Reporting for specific materials, systems and methods

We require information from authors about some types of materials, experimental systems and methods used in many studies. Here, indicate whether each material, system or method listed is relevant to your study. If you are not sure if a list item applies to your research, read the appropriate section before selecting a response.

Materials & experimental systems

n/a	Involved in the study
☒	☐ Antibodies
☒	☐ Eukaryotic cell lines
☒	☐ Palaeontology and archaeology
☒	☐ Animals and other organisms
☒	☐ Clinical data
☒	☐ Dual use research of concern
☒	☐ Plants

Methods

n/a	Involved in the study
☒	☐ ChIP-seq
☒	☐ Flow cytometry
☒	☐ MRI-based neuroimaging

Plants

Seed stocks	*Report on the source of all seed stocks or other plant material used. If applicable, state the seed stock centre and catalogue number. If plant specimens were collected from the field, describe the collection location, date and sampling procedures.*
Novel plant genotypes	*Describe the methods by which all novel plant genotypes were produced. This includes those generated by transgenic approaches, gene editing, chemical/radiation-based mutagenesis and hybridization. For transgenic lines, describe the transformation method, the number of independent lines analyzed and the generation upon which experiments were performed. For gene-edited lines, describe the editor used, the endogenous sequence targeted for editing, the targeting guide RNA sequence (if applicable) and how the editor was applied.*
Authentication	*Describe any authentication procedures for each seed stock used or novel genotype generated. Describe any experiments used to assess the effect of a mutation and, where applicable, how potential secondary effects (e.g. second site T-DNA insertions, mosiacism, off-target gene editing) were examined.*
